# The Reuniens and Rhomboid Nuclei Are Required for Acquisition of Pavlovian Trace Fear Conditioning in Rats

**DOI:** 10.1523/ENEURO.0106-20.2020

**Published:** 2020-06-23

**Authors:** Yu-Ju Lin, Ruei-Jen Chiou, Chun-hui Chang

**Affiliations:** 1 Institute of Systems Neuroscience, National Tsing Hua University, Hsinchu 30013, Taiwan; 2 Department of Anatomy and Cell Biology, School of Medicine, College of Medicine, Taipei Medical University, Taipei 11031, Taiwan

**Keywords:** behavioral pharmacology, nucleus reuniens, rhomboid nucleus, trace fear conditioning

## Abstract

The reuniens (Re) and rhomboid (Rh) nuclei (ReRh) of the midline thalamus interconnects the hippocampus (HPC) and the medial prefrontal cortex (mPFC). Several studies have suggested that the ReRh participates in various cognitive tasks. However, little is known about the contribution of the ReRh in Pavlovian trace fear conditioning, a procedure with a temporal gap between the conditioned stimulus (CS) and the unconditioned stimulus (US), and therefore making it harder for the animals to acquire. Because the HPC and mPFC are involved in trace, but not delay, fear conditioning and given the role of the ReRh in mediating this neurocircuitry, we hypothesized that ReRh inactivation leads to a learning deficit only in trace conditioning. In a series of experiments, we first examined the c-Fos expression in male Long–Evans rats and established that the ReRh was recruited in the encoding, but not the retrieval phase, of fear memory. Next, we performed behavioral pharmacology experiments and found that ReRh inactivation impaired only the acquisition, but not the consolidation or retrieval, of trace fear. However, although the ReRh was recruited during the encoding of delay fear demonstrated by c-Fos results, ReRh inactivation in any phases did not interfere with delay conditioning. Finally, we found that trace fear acquired under ReRh inactivation reprised when the ReRh was brought off-line during retrieval. Together, our data revealed the essential role of the ReRh in a learning task with temporally discontinuous stimuli.

## Significance Statement

The behavior and neurobiology of normal and pathologic fear learning have long been an interest to scientists since malfunction of fear regulation may lead to severe psychiatric disorders. To capture the complex behavior of fear learning, in a rodent model, we studied trace fear conditioning, along with delay procedure, to investigate the recruitment of the reuniens (Re) and rhomboid (Rh) nuclei (ReRh) in a task with temporally discontinuous stimuli. We found that the ReRh is involved during the acquisition of trace fear. However, without a functional ReRh during acquisition, retrieval of trace fear is state-dependent and likely generalized. Our study revealed the essential role of the ReRh in different learning procedures of fear conditioning.

## Introduction

Fear is a vital negative emotion that helps organisms cope with potential dangers, avoid further harm, and increase survival chances (LeDoux, 1996). However, malfunction of fear may lead to mental disorders, such as phobia, panic disorder, and posttraumatic stress disorder (PTSD; Milad et al., 2006; Quirk and Mueller, 2008). Therefore, it is essential to study the psychological processes and neurobiological mechanisms of fear learning. Pavlovian fear conditioning is frequently used to study the neuronal bases of fear learning and memory (Rigoli et al., 2016). In this behavioral procedure, animals learn to associate the initially neutral conditioned stimulus (CS), e.g., a tone, with an aversive unconditioned stimulus (US), e.g., a mild footshock. Different temporal arrangements of the stimuli lead to different behavioral procedures: in delay conditioning, the CSs and USs overlap in presentation and co-terminate, while in trace conditioning, the stimuli are separated in time by a “trace” interval (Pavlov, 1927; Raybuck and Lattal, 2014). Trace fear learning resembles human emotional learning in that human learning sometimes involves a temporal gap. Notably, the insertion of a trace interval affects associative learning (Shors et al., 2000). Subjects usually require more trials to acquire trace fear (Pavlov, 1927; Beylin et al., 2001) and the trace CS-US association is weaker, demonstrated by lower freezing levels following trace conditioning (Raybuck and Lattal, 2014). Indeed, this variant of the procedure recruits profoundly different and higher-order neuronal circuitry. The importance of the hippocampus (HPC) and the medial prefrontal cortex (mPFC) in trace conditioning has been repeatedly examined. Bangasser et al. (2006) demonstrated that hippocampal lesions only impaired trace, but not delay, fear conditioning. Indeed, electrophysiology results showed that hippocampal intrinsic excitability and synaptic plasticity are involved in the acquisition of trace fear conditioning (Song et al., 2012). Gilmartin and Helmstetter (2010) showed that pharmacological inactivation or blockade of NMDA receptor-dependent transmission in the mPFC impaired the acquisition of trace fear conditioning. Moreover, active firing of mPFC neurons (Blum et al., 2006; Gilmartin et al., 2013) and changes in intrinsic excitability of amygdala-projecting mPFC neurons (Song et al., 2015) were demonstrated in trace fear conditioning.

Anatomically, the mPFC does not directly project to the HPC (Laroche et al., 2000; Vertes, 2004), but it can communicate with the HPC through the reuniens (Re) and rhomboid (Rh) nuclei (ReRh) of the midline thalamus (Hoover and Vertes, 2012). Other than the ReRh, the perirhinal cortex (PRC; Witter et al., 2000; Delatour and Witter, 2002) and entorhinal cortex (EC; Burwell and Amaral, 1998; Agster and Burwell, 2009) might also act as relay stations. The ReRh interconnects with the mPFC and the HPC (Vertes et al., 2006) and is necessarily involved in tasks that require coordinated mPFC and HPC activity. Indeed, inactivation of the ReRh caused deficits in a working memory-dependent conditional discrimination task (Hallock et al., 2013), impaired performance in a spatial working memory-specific task (Layfield et al., 2015), and disrupted hippocampal-prefrontal synchrony (Hallock et al., 2016). Relevant to the present study, Ramanathan et al. (2018a) recently demonstrated that inactivation of the ReRh impaired the acquisition of contextual fear memory and led to fear generalization. These studies suggest that the ReRh coordinates the mPFC-HPC network, which is important in the establishment and maintenance of the association between stimuli in various cognitive tasks (Cassel et al., 2013; Dolleman-van der Weel et al., 2019). Previous research speculated that the mPFC may modulate memory specificity via its inputs to the ReRh, which in turn signals to both the HPC and back to the mPFC, making the ReRh a critical node in the mPFC-HPC circuitry (Xu and Südhof, 2013).

Given the role of the ReRh in mPFC-HPC interactions, we sought to determine whether the ReRh is involved in different learning phases of trace fear conditioning and hypothesized that recruitment of the ReRh is necessary only for trace, but not delay, fear conditioning. To address our question, we used immunohistochemical approach to quantify c-Fos expression, followed by behavioral pharmacology experiments, to examine the general recruitment and the determinant role of the ReRh in different learning phases (acquisition, consolidation, and retrieval) of trace fear. We also explored whether there is a state-dependent effect of the ReRh inactivation as suggested by previous literature in contextual fear conditioning (Ramanathan et al., 2018a). Together, our data revealed the importance of the ReRh during the acquisition of trace fear conditioning.

## Materials and Methods

### Subjects

A total of 180 Long–Evans male rats (initially weighing 200–224 g; National Laboratory Animal Center, NARLabs, Taiwan) were used. All animals were individually housed in the animal facility at National Chiao Tung University under professional animal care staff and on-site veterinarian. Animals were maintained on a 12/12 h light/dark cycle with food and water provided *ad libitum*. All animals were handled for at least 5 d (10 s/d) before any experimental procedure. All experimental procedures were performed during the light cycle (7 A.M. to 7 P.M.) and followed the guidelines approved by both of National Tsing Hua University and National Chiao Tung University, Institutional Animal Care and Use Committees (IACUC).

### Surgical procedures

**Figure 1.**
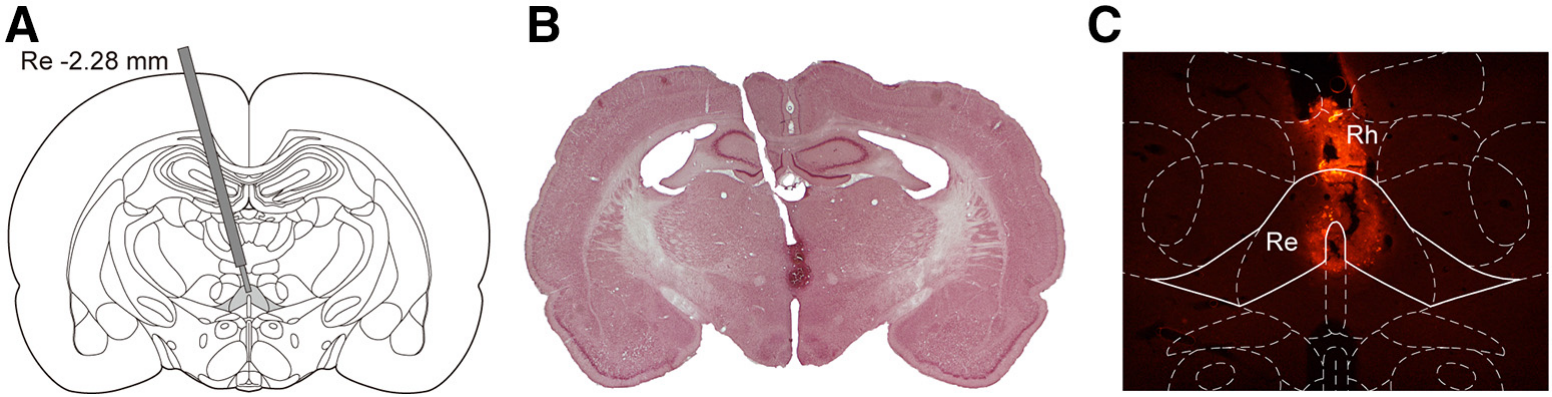
Histology confirmation of this study. ***A***, An illustrative representation showing cannula placement in the ReRh. ***B***, A Nissl-stained coronal section showing the cannula placement in the ReRh. ***C***, A dark-field image showing diffusion of TMR-X muscimol in the ReRh.

Animals were anesthetized with ketamine (80–100 mg/kg) and xylazine (8–10 mg/kg) and then placed in a stereotaxic apparatus (Stoelting). Core body temperature was maintained at 37°C by a temperature-controlled heating pad (CWE). A single 26-gauge stainless steel guide cannula was implanted aiming the Re (relative to bregma: anterior-posterior −2.3 mm, medial-lateral +1.9 mm, and dorsal-ventral −6.5 mm) at a 15° angle to the vertical axis. An example of illustrative representation and its Nissl-stained coronal section are shown in Figure 1*A*,*B*. Three anchor screws were mounted and the headstage was fixed with dental acrylic. Carprofen (5 mg/kg) was subcutaneously injected after surgery and the following 2 d. After surgery, animals were placed in their home cages and monitored until awake. Behavioral procedures were performed at least 5 d after surgeries. In the meantime, dummies (1.0 mm longer than cannula) were changed daily to prevent blockade of the cannulae.

### Drug infusions

Drug infusions were performed at different time points according to our experimental design (experiments 2–5, see below); 33-gauge injectors (extending 1.0 mm beyond the guide cannula) were attached to polyethylene tubes that were connected to Hamilton syringes located on an infusion pump (Harvard Apparatus). Muscimol (0.5 μg/μl; Alfa Aesar) or vehicle (sterile saline) was injected at the rate of 0.25 μl/min for 2 min, followed by another 30 s for drug diffusion. An example of the diffusion of fluorescent-labeled TMR-X muscimol (Life Technologies) in the ReRh is shown in Figure 1*C*. Notably, the inactivation was subtotal and was limited to the injection center of the Re and the above Rh, whereas the more lateral portions of the Re were not affected. The dosage of muscimol was chosen based on earlier studies for similar experimental purposes (Hallock et al., 2013; Layfield et al., 2015). The effect of muscimol lasts ∼3–4 h after injection (Martin, 1991; van Duuren et al., 2007).

### Behavioral apparatus

Four fear conditioning chambers (Med-Associates) were used with two context settings. In Context A, animals were transported to the chambers in cuboids. The doors of the chambers were half open and the chamber lights were on. The fans attached to the chambers were in operation, which also worked as background noise. Additionally, the pans beneath the chambers were filled with 1% acetic acid, providing a distinct odor. In Context B, animals were transported to the chambers in cylinders and covered with black sheets. The lights in the behavioral room were blurred red, which was a dark surrounding for rats. The doors of the chambers were closed and the chamber lights were off. The fans attached to the chambers were off. There were A-frame inserts and acrylic plates above the grids inside the chambers. Additionally, the pans were filled with 1% ammonium.

### Experimental design

#### Experiment 1

Animals were assigned into four groups: TRACE, DELAY, UNPAIRED, and NoCOND. On day 1, each group received different conditioning procedure in Context A. After a 3-min baseline (BL), each group received a ten-trial session of auditory fear conditioning using a 20-s auditory CS (85 dB, 2 kHz) and a 2-s footshock US (1.0 mA) with a 240-s intertrial interval (ITI). For TRACE rats, there was a 30-s trace interval (stimulus-free period) between CSs and USs, whereas for DELAY rats, the CSs always co-terminated with the USs. For UNPAIRED rats, CSs and USs were not paired, with the interval in between randomized ranging from 100 to 120 s (average of 110 s). For NoCOND rats, they received CSs only but no USs. To assess fear encoding, the amount of time spent freezing during the BL and the first 18-s CS (to avoid disturbance by the footshock in the DELAY group) was measured and analyzed. On day 2, all rats received three 20-s CS during the test session in Context B with an ITI of 60 s. The test was performed in a different context from conditioning to minimize contextual fear, and only three CSs were given to ensure that c-Fos expression corresponded to fear retrieval, but not fear extinction (Bouton, 2004). To assess fear retrieval, the amount of time spent freezing during the BL and during the 20-s CS was measured and analyzed.

#### Experiments 2–4

Three independent experiments with drug manipulation at different time points were done. For each experiment, a mixed design with between-subject factors of “drug” [muscimol (M) and vehicle (V)/or control (C)] and “group” (TRACE and DELAY) and within-subject factor of “trials” was used, yielding a total of four groups: M-TRACE, V(C)-TRACE, M-DELAY, and V(C)-DELAY. Drug infusions were performed according to the experimental design: immediately before conditioning (experiment 2), after conditioning (experiment 3), or immediately before retrieval test (experiment 4). The day before the behavioral experiment, the animals were accustomed to the sound of the infusion pump for 2 min. On day 1, animals were assigned into two groups: trace conditioning (TRACE) and delay conditioning (DELAY). The procedures were the same as in experiment 1 and were performed in Context A. On day 2, all animals received a ten 20-s CS test session in Context B with the ITI of 60 s to assess fear memory.

#### Experiment 5

Animals were assigned into three groups: SAL-SAL, MUS-SAL, and MUS-MUS, and received drug infusions immediately before conditioning and immediately before retrieval test, accordingly. The day before the behavioral experiment, the animals were accustomed to the sound of the infusion pump for 2 min. On day 1, all animals underwent trace fear conditioning procedure the same as described in experiment 1 in Context A. On day 2, all animals received a 10 20-s CS test session in Context B with the ITI of 60 s to assess fear memory.

### Immunohistochemistry

In experiment 1, half of the animals were killed for tissue processing after conditioning, while the other half tested for memory retrieval and then killed after retrieval test for tissue processing. To capture the time window of maximal c-Fos expression, the animals were killed 90 min after the behavioral sessions (conditioning or retrieval test). All animals were deeply anesthetized with CO_2_. Incisions were made within 30 s after animals left the CO_2_ chamber and animals were completely unresponsive during the entire perfusion procedure. To optimize the perfusion, a hemostat was used to clamp the descending aorta. All animals were perfused transcardially with 100 ml of saline followed by 100 ml of 4% paraformaldehyde (PFA) in 0.1 m PBS, serving as a fixative. Brains were removed, postfixed at least 2 h, cryoprotected in 25% sucrose solution for 24–72 h, and then sectioned coronally (35 μm). We performed c-Fos visualization with the standard immunohistochemical procedure. In short, free-floating tissue sections were incubated overnight in a buffer solution with mouse anti-c-Fos antibody (Santa Cruz, catalog #sc-271243, 1:200). The following day after rinsing with buffer several times, sections were incubated in biotinylated donkey anti-mouse IgG (Jackson ImmunoResearch, code #715-065-151, 1:500) solution, and then Vectastatin ABC Elite reagents (Vector Laboratories), followed by a solution of nickel sulfate and diaminobenzidine (DAB) with hydrogen peroxide to produce a blue-black reaction product within the nucleus of c-Fos+ neurons.

### Histology

To verify cannula placements in experiments 2–5, brains were collected after the retrieval test and fixed with 8% PFA in PBS. After 48 h, the brains were transferred to 25% sucrose in 0.1 m PBS until saturated. Brains were then sectioned coronally (60 μm). The slices were mounted onto subbed slides for standard Nissl staining to confirm the injection sites.

### Statistical analysis

For experiment 1, our analyses focused on the Re only. All c-Fos+ neurons within the Re were manually counted with microscopy at three coronal levels: −1.56, −2.52, and −3.48 mm, relative to bregma. The counts in each group were submitted to one-way ANOVA. All significant F-ratios were reported, and after significant F-ratios were obtained, Tukey’s HSD *post hoc* analyses were performed.

All behavioral procedures were recorded (Video Freeze, Med-Associates; sampling at 0.2 s), and freezing was defined as consecutively observed movements for 1 s below the motion threshold (program set at 100). Freezing behavior was measured continuously during all of the behavioral sessions. The percentage of total observations in which freezing occurred at BL and during CSs was calculated. These values were submitted to repeated measures of ANOVA (RM ANOVA). All significant F-ratios were reported, and after significant F ratios were obtained, Tukey’s HSD *post hoc* analyses were performed.

For all ANOVAs, effect sizes were reported as partial η^2^ (η^2^_p_; Fritz et al., 2012; Lakens, 2013). As an effect size, η^2^_p_ values > 0.01, 0.06, and 0.14 are generally interpreted as small, medium, and large effects, respectively (Richardson, 2011). All data were calculated using SPSS (IBM) and presented as mean ± SEM.

## Results

### Experiment 1: Re is generally recruited during the encoding, but not the retrieval, of trace and delay fear memory

**Figure 2.**
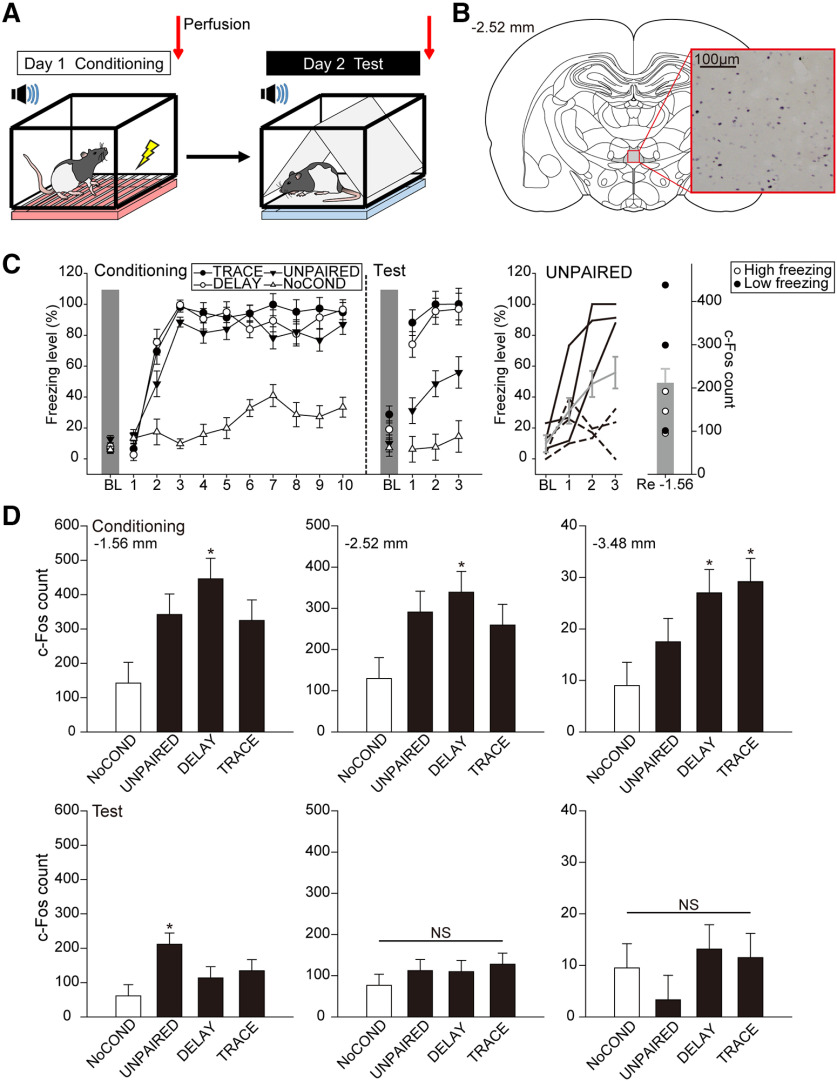
Re neurons were generally recruited in the encoding phase of trace and delay fear conditioning. ***A***, Animals underwent a 2-d behavioral experiment, in which half of the animals in each group were killed after conditioning and the rest after retrieval test. ***B***, c-Fos+ expression in Re neurons (red square). c-Fos signals were visualized with blue/black reaction products within the nucleus of activated neurons. ***C***, The freezing behavior of TRACE, DELAY, UNPAIRED, and NoCOND groups. Freezing levels of the conditioning (*n* = 12 per group) and test trials (*n* = 6 per group) are presented in left panel, respectively. The middle panel shows the averaged freezing levels of the UNPAIRED group (gray line with SEM) and the divergent high (solid lines) or low (dashed lines) freezing levels of each animal during retrieval test. The right panel shows the average c-Fos+ count of the UNPAIRED group (gray bar with SEM) and the counts of high freezing (open circles; behaviors shown in solid lines) and low freezing (filled circles; behaviors shown in dashed lines) animals based on their behavior on trial 3 (*n* = 3 each). ***D***, Group differences in c-Fos+ counts of the Re after conditioning (upper panel) and test (lower panel); *p < 0.05. NS, not significant.

In this experiment, we first established whether the Re is recruited in delay and trace fear conditioning using a 2-d procedure (Fig. 2*A*). A total of 48 animals were used. On day 1, animals underwent the conditioning procedure according to their assigned groups (*n* = 12 per group), and then half of the animals in each group was killed. On day 2, the remaining of the animals (*n* = 6 per group) were tested for fear retrieval in a different context to minimize contextual fear and then killed. The tissues were processed to visualize c-Fos expression in the Re as an index of activation during encoding (day 1) and retrieval (day 2) of fear memory.

The behavioral results are summarized in Figure 2*C*, left panel. On day 1, TRACE, DELAY, and UNPAIRED groups exhibited a robust increase in freezing levels toward later trials, whereas NoCOND group exhibited low freezing levels throughout the conditioning session. There were significant main effects of group [*F*_(3,44)_ = 72.24, *p* < 0.001, η^2^_p_ = 0.83] and trials [*F*_(10,440)_ = 86.34, *p* < 0.001, η^2^_p_ = 0.66], and a significant interaction between group and trials [*F*_(30,440)_ = 7.07, *p* < 0.001, η^2^_p_ = 0.33]. *Post hoc* comparisons suggested that freezing levels in TRACE, DELAY, and UNPAIRED groups were significantly higher compared with NoCOND group (all *p*s < 0.001). Moreover, there was no statistical difference in freezing levels between TRACE and DELAY groups (*p* = 0.89), indicating equivalent acquisition under delay and trace procedures during conditioning.

On day 2, the animals were tested for their fear to the tones. Both DELAY and TRACE groups demonstrated high freezing levels during the CSs, while UNPAIRED and NoCOND groups demonstrated low freezing levels. There were significant main effects of group [*F*_(3,20)_ = 29.06, *p* < 0.001, η^2^_p_ = 0.81] and trials [*F*_(3,60)_ = 49.70, *p* < 0.001, η^2^_p_ = 0.71], and a significant interaction between group and trials [*F*_(9,60)_ = 6.33, *p* < 0.001, η^2^_p_ = 0.49]. *Post hoc* comparisons suggested that there was no statistical difference in freezing levels between the TRACE and DELAY groups (*p* = 0.80), which were both significantly higher than UNPAIRED or NoCOND group (all *p*s < 0.05), indicating equivalent learning achieved by the two groups. The result was inconsistent with previous literature, i.e., generally lower freezing levels were observed following trace conditioning compared with delay conditioning (Raybuck and Lattal, 2014). Interestingly, the freezing level of the UNPAIRED group was significantly higher compared with NoCOND group (*p* < 0.05), yet significantly lower compared with DELAY and TRACE groups (both *p*s < 0.05). When we further looked into the freezing level of each animal in this group (Fig. 2*C*, middle panel), we found that the rats displayed two distinct behavioral patterns that half of them showed high fear (solid lines, *n* = 3), whereas the other half showed low fear (dashed lines, *n* = 3), to tones.

An example of c-Fos expression in the Re is shown in Figure 2*B*. During the encoding phase (Fig. 2*D*, upper panel) of fear, ANOVA revealed significant main effects of group in all levels examined (relative to bregma, −1.56, −2.52, and −3.48 mm) [−1.56 mm, *F*_(3,20)_ = 4.40, *p* = 0.016, η^2^_p_ = 0.40; −2.52 mm, *F*_(3,20)_ = 3.15, *p* = 0.048, η^2^_p_ = 0.32; −3.48 mm, *F*_(3,20)_ = 4.20, *p* = 0.019, η^2^_p_ = 0.39]. *Post hoc* comparisons suggested that compared with NoCOND group, the numbers of c-Fos+ neurons were consistently higher in DELAY group in the Re (all *p*s < 0.05), while the c-Fos+ counts of TRACE group were significantly higher at the level of −3.48 mm (*p* < 0.05). However, although c-Fos+ counts also increased in the UNPAIRED group compared with NoCOND group, the differences did not reach significance in any of the levels we examined (all *p*s > 0.05). During the retrieval phase (Fig. 2*D*, lower panel) of fear, ANOVA revealed a significant main effect of group [*F*_(3,20)_ = 3.68, *p* = 0.029, η^2^_p_ = 0.36] only at level −1.56 mm. *Post hoc* comparisons suggested that compared with NoCOND group, the significant difference is with the UNPAIRED group only (*p* < 0.05). When we further looked into the c-Fos activation level based on their freezing behavior in this group (Fig. 2*C*, middle panel), we found that the three animals with high freezing levels had low c-Fos activation (Fig. 2*C*, right panel, open circles; c-Fos count =192, 146, and 95), consistent with those of DELAY and TRACE groups. On the contrary, the ones with low freezing levels were more likely to have high c-Fos activation (Fig. 2*C*, right panel, filled circles; c-Fos count = 438, 299, and 101) in the Re, a result suggesting that this brain structure was recruited when the animals displayed no fear at the behavioral level. Together, these results revealed critical insights into the recruitment of the Re neurons during the encoding, but not the retrieval phase, of delay and trace fear memory.

### Experiment 2: ReRh is necessary for the acquisition phase of trace fear memory

In experiment 1, we demonstrated evidences that the Re is recruited during the encoding of delay and trace fear memory. However, a recent study indicated that the ReRh is also important for the encoding of contextual fear (Ramanathan et al., 2018a). Thus, we cannot rule out the possibility that the neurons were activated because of contextual fear, rather than CS-induced fear. To address the question, we designed a series of behavioral pharmacology experiments (experiments 2–4) to determine whether the ReRh is necessarily recruited in delay and trace conditioning.

**Figure 3.**
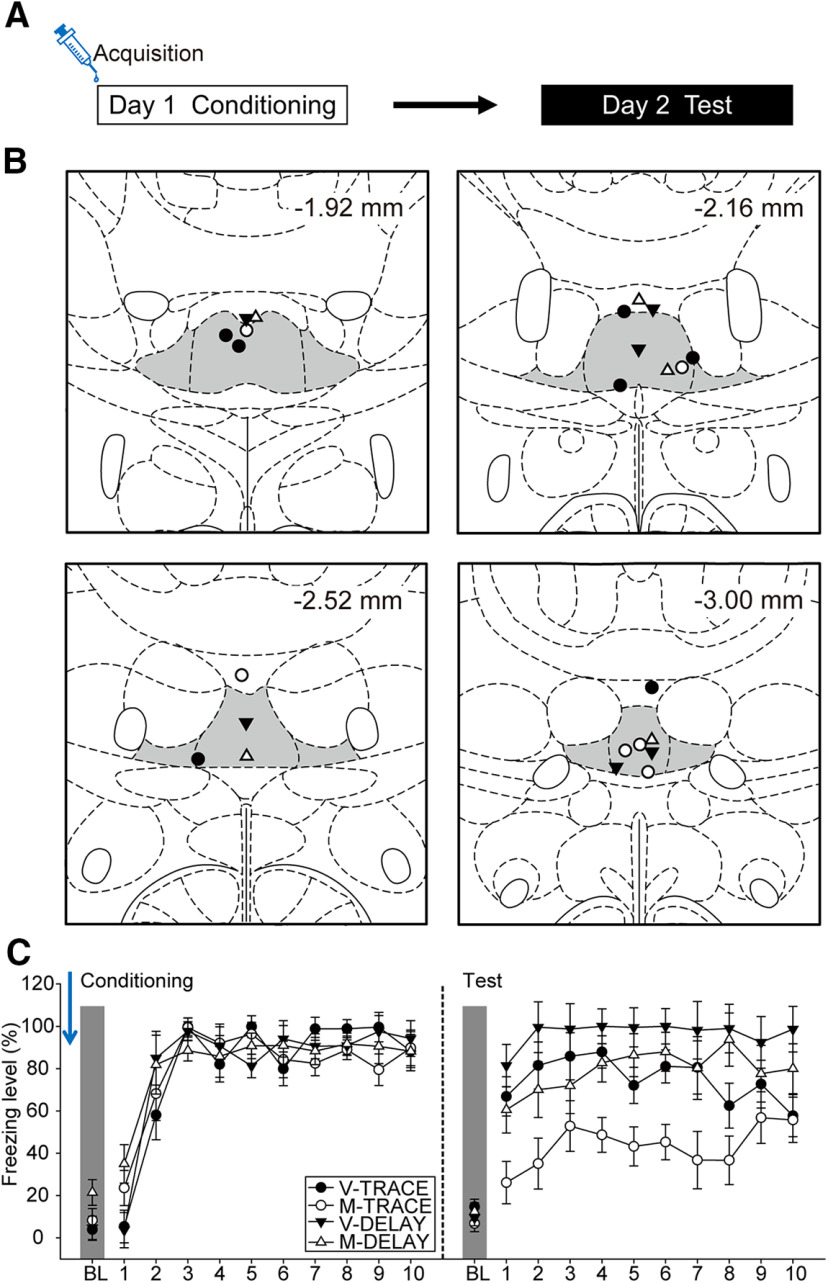
The ReRh was critically involved during the acquisition of trace fear conditioning. ***A***, Experimental design. Animals underwent a 2-d behavioral experiment, in which the animals received drug infusion immediately before conditioning (day 1). ***B***, Injector tip placements of all the animals included in data analyses at levels −1.92, −2.16, −2.52, and −3.00 mm posterior relative to bregma. The gray areas marked in the atlas are the Re and ventral Re. ***C***, The freezing behavior of V-TRACE (*n* = 7), M-TRACE (*n* = 6), V-DELAY (*n* = 6), and M-DELAY groups (*n* = 5) when injections were performed before conditioning.

In this experiment, we examined the determinant role of the ReRh during the acquisition phase of trace fear (Fig. 3*A*). The placements of the injector tips for all the animals included in data analyses are summarized in Figure 3*B*. Of the initial 32 rats that underwent surgeries, one was excluded due to death during surgery and seven due to cannula misplacements, leading to the following final group sizes: V-TRACE (*n* = 7), M-TRACE (*n* = 6), V-DELAY (*n* = 6), and M-DELAY (*n* = 5).

On day 1, the animals underwent the conditioning procedure (Fig. 3*C*, left panel). All animals showed an increase in freezing levels as the trials proceeded. There was a significant main effect of trials [*F*_(10,200)_ = 81.75, *p* < 0.001, η^2^_p_ = 0.80] and a significant two-way interaction between drug and trials [*F*_(10,200)_ = 2.22, *p* = 0.018, η^2^_p_ = 0.10]. Under the influence of muscimol, these animals had slightly higher freezing levels compared with controls during BL and the first CS. However, at the last trial of conditioning, there was no statistical difference in freezing levels among groups (all *p*s > 0.05).

On day 2, all animals were tested for their acquisition of delay or trace conditioning under ReRh inactivation. (Fig. 3*C*, right panel). All animals demonstrated fear responses to the tones. There were significant main effects of group [*F*_(1,20)_ = 20.68, *p* < 0.001, η^2^_p_ = 0.51], drug [*F*_(1,20)_ = 15.30, *p* = 0.001, η^2^_p_ = 0.43], and trials [*F*_(10,200)_ = 18.01, *p* < 0.001, η^2^_p_ = 0.47]. However, the lack of significant two-way interaction between group and drug [*F*_(1,20)_ = 1.34, *p* = 0.26, η^2^_p_ = 0.06] suggested that both delay and trace procedures may have been affected by ReRh inactivation during acquisition. This result was inconsistent with the earlier literature, i.e., inactivation of the ReRh did not impair the acquisition of delay fear conditioning (Ramanathan et al., 2018a). We therefore did single-factor analysis for DELAY and TRACE groups to further examine the respective drug effects. For DELAY group, there was a significant main effect of trials [*F*_(10,90)_ = 24.61, *p* < 0.001, η^2^_p_ = 0.73], but only a marginal effect of drug [*F*_(1,9)_ = 4.23, *p* = 0.07, η^2^_p_ = 0.32]. For TRACE group, there were significant main effects of drug [*F*_(1,11)_ = 12.29, *p* = 0.005, η^2^_p_ = 0.53] and trials [*F*_(10,110)_ = 4.76, *p* < 0.001, η^2^_p_ = 0.30]. Together, our data suggested that ReRh inactivation during conditioning impaired fear encoding, and this effect was in majority attributed to the TRACE group.

### Experiment 3: ReRh is not necessary for the consolidation phase of trace fear memory

**Figure 4.**
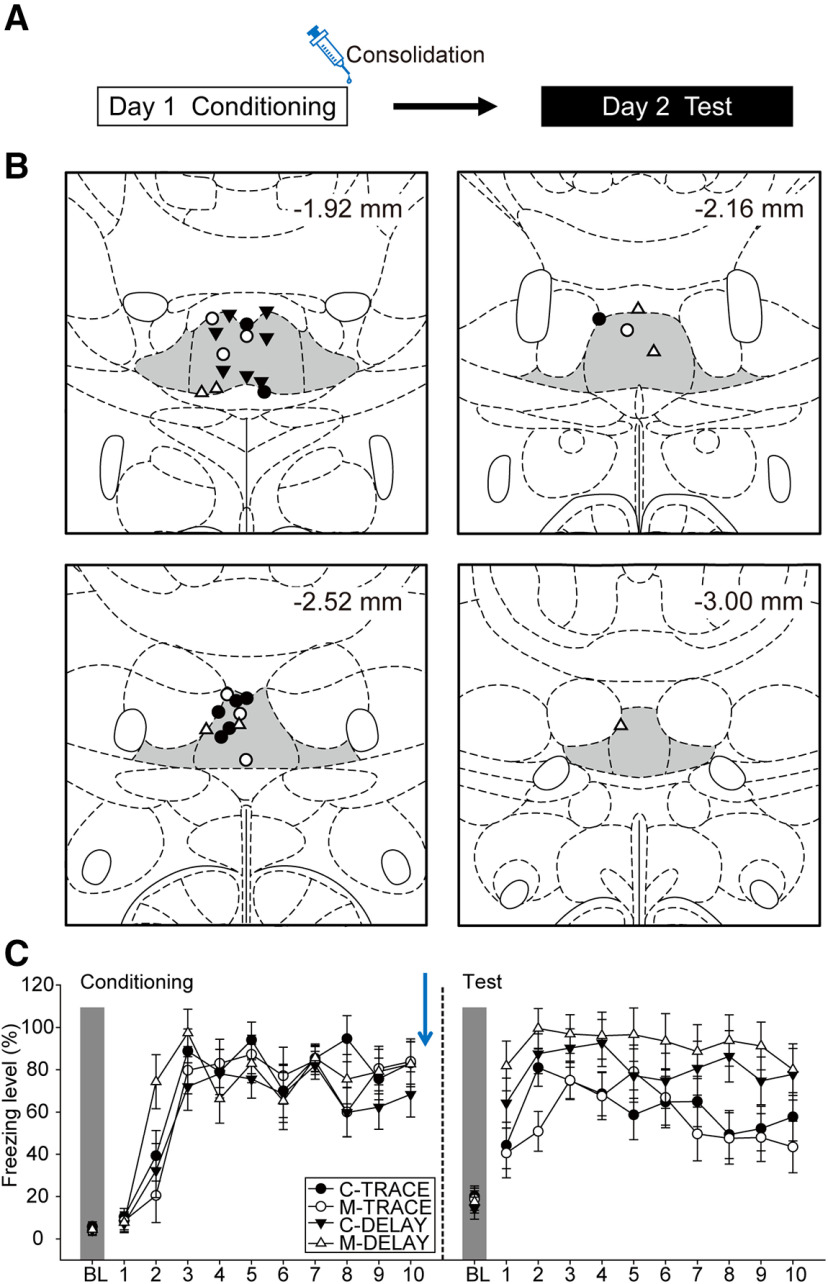
The ReRh was not involved during the consolidation of trace fear conditioning. ***A***, Experimental design. Animals underwent a 2-d behavioral experiment, in which the animals received drug infusion immediately after conditioning (day 1). ***B***, Injector tip placements of all the animals included in data analyses at levels −1.92, −2.16, −2.52, and −3.00 mm posterior relative to bregma. The gray areas marked in the atlas are the Re and ventral Re. ***C***, The freezing behavior of C-TRACE (*n* = 8), M-TRACE (*n* = 7), C-DELAY (*n* = 7), and M-DELAY groups (*n* = 7) when injections were performed after conditioning.

In this experiment, we examined the role of the ReRh during the consolidation phase of trace fear (Fig. 4*A*). The placements of the injector tips for all the animals included in data analyses are summarized in Figure 4*B*. Of the initial 32 rats that underwent surgeries, one was excluded due to death during surgery and two due to cannula misplacements, leading to the following final group sizes: C-TRACE (*n* = 8), M-TRACE (*n* = 7), C-DELAY (*n* = 7), and M-DELAY (*n* = 7).

On day 1, the animals underwent the conditioning procedure (Fig. 4*C*, left panel). All animals showed an increase in freezing levels as the trials proceeded. There was a significant main effect of trials [*F*_(10,250)_ = 51.77, *p* < 0.001, η^2^_p_ = 0.67] and a significant three-way interaction among group, drug, and trials [*F*_(10,250)_ = 2.09, *p* = 0.026, η^2^_p_ = 0.08]. There were fluctuations in freezing levels among the four groups across trials. However, at the last trial of conditioning, there was no statistical difference in freezing levels among them (all *p*s > 0.05). Because newly acquired memory is less stable and may be prone to any interference during consolidation (McGaugh, 2000), it is possible that potential behavioral effects may result from the infusion itself. To rule out this possibility, the controls were further divided into vehicle (DELAY, *n* = 4; TRACE, *n* = 4) and home (DELAY, *n* = 3; TRACE, *n* = 4). Animals in the vehicle group underwent vehicle infusion right after the conditioning procedure, whereas those in the home group went back to home cages after the conditioning procedure without further manipulation.

On day 2, all animals were tested for their consolidation of delay or trace conditioning under ReRh inactivation. We first double-checked the behavioral performances between the vehicle and the home groups. Because there were no statistical differences in any of the main effect or interactions regarding postconditioning vehicle infusion during retrieval test (all *p*s ≥ 0.05), the two groups were merged as respective control groups (C-TRACE and C-DELAY) in the following analyses. All animals showed high levels of CS-elicited freezing behavior after tone onset and slightly decreased toward later trials (Fig. 4*C*, right panel). There were significant main effects of group [*F*_(1,25)_ = 7.74, *p* = 0.01, η^2^_p_ = 0.24] and trials [*F*_(10,250)_ = 27.12, *p* < 0.001, η^2^_p_ = 0.52], and a significant two-way interaction between group and trials [*F*_(10,250)_ = 2.67, *p* = 0.004, η^2^_p_ = 0.10]. However, there were no statistical differences in the main effect of drug [*F*_(1,25)_ < 1, η^2^_p_ = 0.01], two-way interactions between drug and group [*F*_(1,25)_ < 1, η^2^_p_ = 0.03] or drug and trials [*F*_(10,250)_ = 1.30, *p* = 0.23, η^2^_p_ = 0.05], or three-way interaction among drug, group, and trials [*F*_(10,250)_ < 1, η^2^_p_ = 0.03]. The lack of drug main effect and its interactions suggested that postconditioning inactivation of the ReRh did not affect the consolidation of trace fear memory. The higher freezing levels in DELAY group compared with TRACE group and the faster decline in freezing levels toward later trials in TRACE group supported the idea that the CS-US association is weaker in trace fear conditioning (Raybuck and Lattal, 2014).

### Experiment 4: ReRh is not necessary for the retrieval phase of trace fear memory

**Figure 5.**
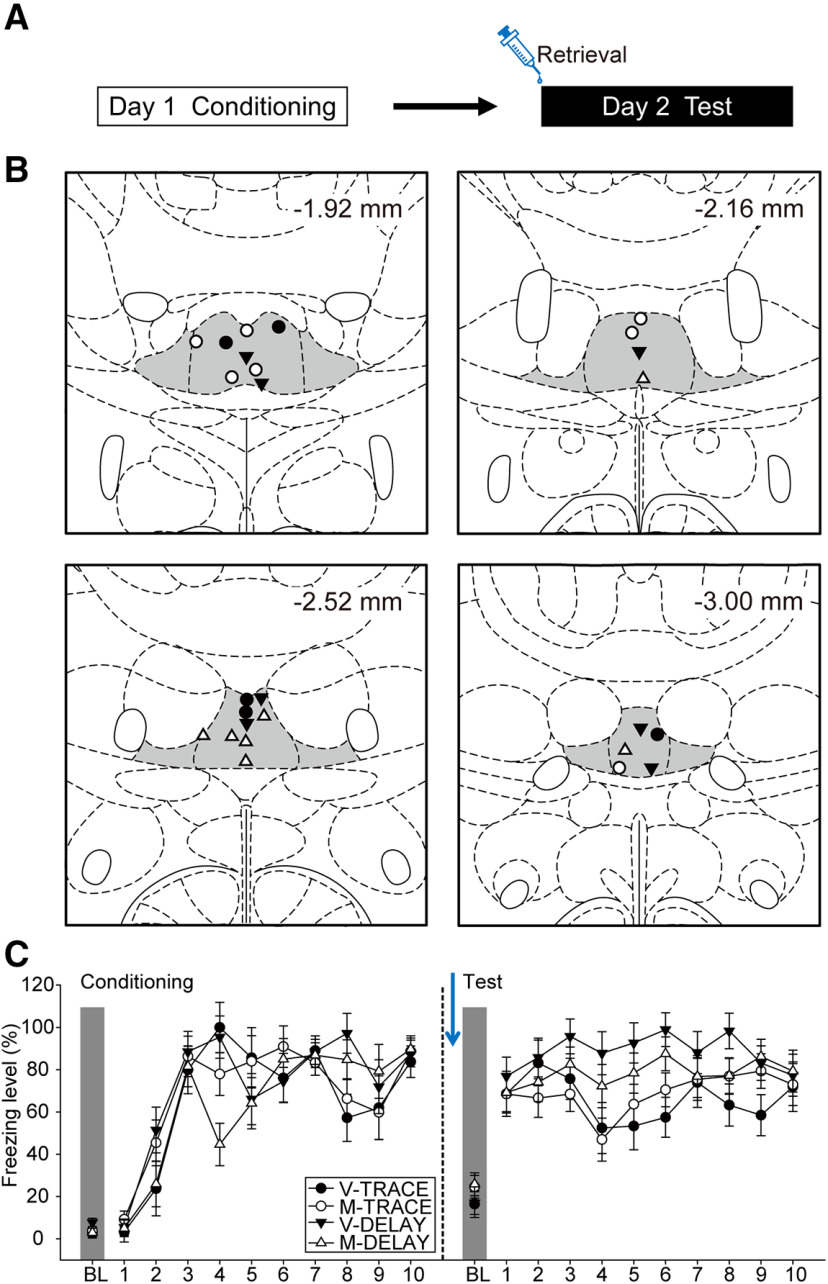
The ReRh was not involved during the retrieval of trace fear conditioning. ***A***, Experimental design. Animals underwent a 2-d behavioral experiment, in which the animals received drug infusion immediately before test (day 2). ***B***, Injector tip placements of all the animals included in data analyses at levels −1.92, −2.16, −2.52, and −3.00 mm posterior relative to bregma. The gray areas marked in the atlas are the Re and ventral Re. ***C***, The freezing behavior of V-TRACE (*n* = 5), M-TRACE (*n* = 7), V-DELAY (*n* = 7), and M-DELAY groups (*n* = 7) when injections were performed before retrieval test.

In this experiment, we examined the role of the ReRh during the retrieval phase of trace fear (Fig. 5*A*). The placements of the injector tips for all the animals included in data analyses are summarized in Figure 5*B*. Of the initial 32 rats that underwent surgeries, one was excluded due to death during surgery and five due to cannula misplacements, leading to the following final group sizes: V-TRACE (*n* = 5), M-TRACE (*n* = 7), V-DELAY (*n* = 7), and M-DELAY (*n* = 7).

On day 1, the animals underwent the conditioning procedure (Fig. 5*C*, left panel). All animals showed an increase in freezing levels as the trials proceeded. There was a significant main effect of trials [*F*_(10,220)_ = 49.85, *p* < 0.001, η^2^_p_ = 0.70] and a significant two-way interaction between group and trials [*F*_(10,220)_ = 2.30, *p* = 0.014, η^2^_p_ = 0.10]. Although there were fluctuations in freezing levels between delay or trace procedures across trials, there was no statistical difference in freezing levels among groups at the last trial of conditioning (all *p*s > 0.05).

On day 2, all animals were tested for their retrieval of delay or trace conditioning under ReRh inactivation. All animals showed high levels of CS-elicited freezing behavior after tone onset and the freezing levels maintained high throughout the test session (Fig. 5*C*, right panel). There were significant main effects of group [*F*_(1,22)_ = 5.36, *p* = 0.03, η^2^_p_ = 0.20] and trials [*F*_(10,220)_ = 23.21, *p* < 0.001, η^2^_p_ = 0.51], and a significant two-way interaction between group and trials [*F*_(10,220)_ = 2.27, *p* = 0.015, η^2^_p_ = 0.10]. However, there were no statistical differences in the main effect of drug [*F*_(1,22)_ < 1, η^2^_p_ = 0.01], two-way interactions between drug and group [*F*_(1,22)_ < 1, η^2^_p_ = 0.04] or drug and trials [*F*_(10,220)_ = 1.25, *p* = 0.26, η^2^_p_ = 0.05], or three-way interaction among drug, group, and trials [*F*_(10,220)_ < 1, η^2^_p_ = 0.03]. The lack of drug main effect and its interactions suggested that pre-test inactivation of the ReRh did not affect the retrieval of trace fear memory. The higher freezing levels in DELAY group compared with TRACE group was consistent with the results of experiment 3 showing that CS-US association was weaker in trace fear conditioning.

To sum up experiments 2–4, our results revealed that inactivation of the ReRh impaired the acquisition, but not the consolidation or retrieval, of trace fear. This finding is in line with the results in experiment 1, further supporting its critical role in the encoding of trace fear. However, pharmacological inactivation of the ReRh at any of the phases we examined did not interfere with delay fear conditioning.

### Experiment 5: retrieval of trace fear acquired under ReRh inactivation reprised under ReRh inactivation

**Figure 6.**
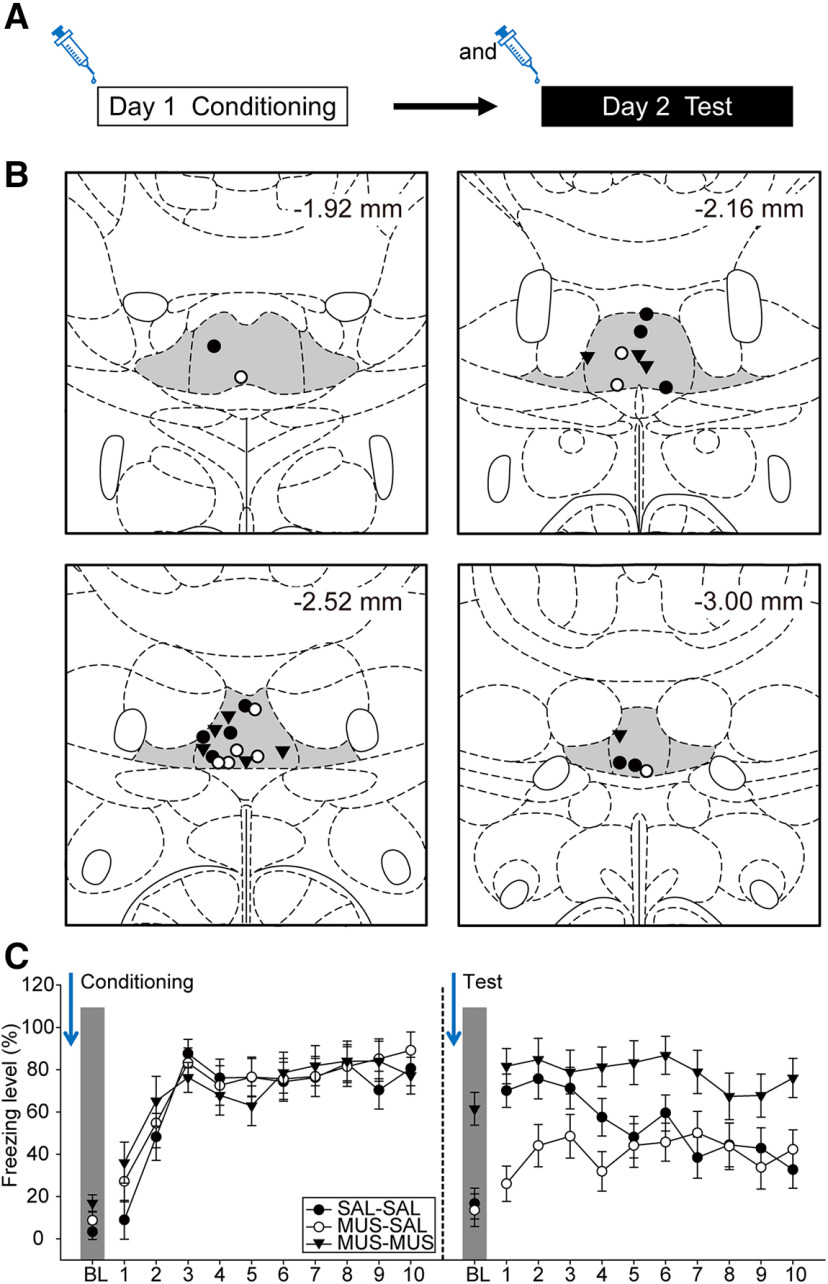
Retrieval of trace fear acquired under ReRh inactivation reprised under ReRh inactivation. ***A***, Experimental design. Animals underwent a 2-d behavioral experiment, in which the animals received drug infusion immediately before both conditioning and retrieval test. ***B***, Injector tip placements of all the animals included in data analyses at levels −1.92, −2.16, −2.52, and −3.00 mm posterior relative to bregma. The gray areas marked in the atlas are the Re and ventral Re. ***C***, The freezing behavior of SAL-SAL (*n* = 10), MUS-SAL (*n* = 9), and MUS-MUS groups (*n* = 9) when the injections were performed before both conditioning and retrieval test.

In experiment 2, we established that the ReRh is critically involved in the acquisition of trace fear. However, earlier literature suggested that retrieval of contextual fear acquired under ReRh inactivation is state dependent (Ramanathan et al., 2018a). To examine whether this is also true for trace fear, we brought the ReRh off-line with muscimol throughout the entire process (Fig. 6*A*). We reasoned that if the trace fear memory acquired under ReRh inactivation is indeed state dependent, then fear to the tones would reprise under ReRh inactivation during retrieval test in this experiment. As our results in experiment 4 indicated that the ReRh is not necessary for the retrieval phase of trace fear memory, SAL-MUS group was not included. The placements of the injector tips for all the animals included in data analyses are summarized in Figure 6*B*. Of the initial 36 rats that underwent surgeries, two were excluded due to death during surgery and six due to cannula misplacements, leading to the following final group sizes: SAL-SAL (*n* = 10), MUS-SAL (*n* = 9), and MUS-MUS (*n* = 9).

On day 1, all animals showed an increase in freezing levels toward later trials during trace conditioning regardless of the injections (Fig. 6*C*, left panel). This is consistent with our results in experiment 2, indicating that ReRh inactivation did not interfere with fear expression. There was a significant main effect of trials [*F*_(10,250)_ = 46.15, *p* < 0.001, η^2^_p_ = 0.65]. However, there were no significant main effect of group [*F*_(2,25)_ < 1, η^2^_p_ = 0.01] or two-way interaction between group and trials [*F*_(20,250)_ = 1.17, *p* = 0.28, η^2^_p_ = 0.09]. All groups reached equivalent high levels of freezing at the last trial (all *p*s > 0.05).

On day 2, we replicated our findings in experiment 2 that compared with SAL-SAL group, MUS-SAL animals demonstrated impaired acquisition of trace fear. Importantly, freezing levels of MUS-MUS group remained high throughout the test session from BL to the last trial (Fig. 6*C*, right panel). There were significant main effects of group [*F*_(2,25)_ = 8.53, *p* = 0.001, η^2^_p_ = 0.41] and trials [*F*_(10,250)_ = 6.46, *p* < 0.001, η^2^_p_ = 0.21], and a significant interaction between group and trials [*F*_(20,250)_ = 1.93, *p* = 0.011, η^2^_p_ = 0.13]. Compared with SAL-SAL group, *post hoc* comparisons suggested that freezing levels of MUS-SAL group were significantly lower in trial 1 (*p* < 0.05) and marginally lower in trial 2 (*p* = 0.076), whereas freezing levels of MUS-MUS group were significantly higher in BL, trial 7, and trial 10 (all *p*s < 0.05). Together, our results suggested that there was a state-dependent retrieval of trace memory that fear acquired under ReRh inactivation only reprised under ReRh inactivation. However, the high levels of BL freezing in the MUS-MUS group and its slower decline in amplitude toward later trials compared with SAL-SAL group also suggested that the fear was generalized.

## Discussion

In the c-Fos visualization experiment, across the antero-posterior axis we examined, the Re was generally recruited in the encoding of delay fear conditioning, whereas the posterior Re was recruited in the encoding of trace fear conditioning. Pharmacological inactivation of the ReRh at different time points further illustrated that the ReRh was crucially involved in the acquisition phase specifically of trace fear. Additionally, trace memory acquired under ReRh inactivation only reprised during ReRh inactivation. In conclusion, our results uncovered a critical role of the ReRh in trace learning at the behavioral level.

A growing body of anatomic, physiological, and behavioral literature has been conducted on the role of the ReRh in behavior and cognition (Cassel et al., 2013; Dolleman-van der Weel et al., 2019). ReRh lesions impaired the spatial representation encoded by hippocampal CA1 place cells by reducing the place field stability and firing variability, indicating the involvement of the ReRh in spatial cognition (Cholvin et al., 2018). Others reported that the ReRh is recruited in navigation by participating in the neurocircuitry involved in the representation of goal-directed routes and mediating the intended movement of the animal (Ito et al., 2015, 2018). Additionally, the ReRh is critical for fear extinction (Ramanathan et al., 2018b), a procedure that involves the HPC in the encoding phase and context-dependent expression of fear after extinction (Maren et al., 2013). Overall, these studies unveil the indispensable character the ReRh plays in hippocampal-dependent memory and tasks. Moreover, the ReRh is involved in cognitive functions that recruit the mPFC-HPC circuitry, such as spatial memory (Loureiro et al., 2012; Mei et al., 2018; Klein et al., 2019), spatial working memory (Layfield et al., 2015; Viena et al., 2018), recognition memory (Barker and Warburton, 2018), sequential memory (Jayachandran et al., 2019), and contextual fear memory (Ramanathan et al., 2018a; Quet et al., 2020).

Despite the abundance of research investigating the role of the ReRh on different demands, studies regarding the tasks that rely on association of stimuli with temporal discontinuity in training, such as trace conditioning, were sparse. Previous studies using pharmacological approaches indicated that trace fear learning was impaired by pre-conditioning unilateral (Gilmartin et al., 2012) or bilateral inactivation of the HPC (Esclassan et al., 2009), as well as bilateral inactivation of the mPFC (Gilmartin and Helmstetter, 2010). Based on these findings, it is speculated that the ReRh serves as a critical node in the information flow from the mPFC to the HPC in trace fear conditioning. Indeed, here we demonstrated that the ReRh is specifically involved in the acquisition phase of trace fear conditioning, supporting its vital role in the encoding of CS-US temporal relationship.

The participation of the ReRh during the acquisition phase of behavioral tasks has been repeatedly demonstrated. For example, inactivation of the ReRh impaired the acquisition of contextual conditioning (Ramanathan et al., 2018a). A recent study using lesion and pharmacology methods revealed the importance of the ReRh in the establishment of depression and its underlying neuromorphological and endocrine effects (Kafetzopoulos et al., 2018). Additionally, optogenetic inhibition during the sample phase, but not the delay or choice phase, significantly decreased choice accuracy in spatial working memory task (Maisson et al., 2018). These earlier results, together with our current findings, point to the convergent recruitment of the mPFC-HPC circuitry in cognitive tasks and emotional processing, in which the ReRh is important in mediating the information exchange during the initial encoding (Hoover and Vertes, 2012). On the contrary, the results are inconsistent regarding its role during the retrieval of memory. For example, ReRh inactivation impaired spatial working memory performance in a delay-dependent manner (Layfield et al., 2015). Using an hM4Di synaptic-silencing approach, it has been shown that the mPFC-Re projection regulates sequential memory retrieval (Jayachandran et al., 2019). On the other hand, the ReRh is not involved in the retrieval process of recent spatial memory (Loureiro et al., 2012) and contextual memory (Ramanathan et al., 2018a). In our study, there were no behavioral effects of ReRh inactivation on consolidation (experiment 3) or retrieval (experiment 4) of trace fear. However, because ten CS-US trials were administered during conditioning in our series of experiments, in which the freezing levels reached a plateau after trial 3, we cannot rule out the possibility that the lack of behavioral effects was due to over-training. It is also worth noticing that in rats, memories become resilient to interferences and stabilized 6 h after training (McGaugh, 1966). The “early phase” of memory consolidation corresponds to synaptic consolidation, which is mediated by intracellular molecular mechanisms and involves changes in the synaptic connections. On the other hand, the “late phase” consolidation correspond to systems consolidation, which requires the reorganization of the memory trace throughout systems level (Dudai, 2004). Immediate postconditioning inactivation of the ReRh (experiment 3) interfered with only the early phase of memory consolidation, which is within hours when long-term potentiation (LTP) is independent of protein synthesis (McGaugh, 2000). The effect of ReRh inactivation on trace fear conditioning during late phase consolidation, which lasts several days and requires gene expression (Abel and Lattal, 2001; Lin et al., 2003) and protein synthesis (Schafe et al., 2000; Santini et al., 2004), is beyond the scope of the present study. Notably, Sierra et al., recently showed that lidocaine-induced inhibition of the Re inhibited LTP induction in the CA1-anterior cingulate cortex pathways (Sierra et al., 2017). Indeed, ReRh lesion impaired remote contextual memory (Quet et al., 2020) and spatial memory (Loureiro et al., 2012; Klein et al., 2019). Together, these studies provide evidence of the role of the ReRh in mediating systems consolidation and memory persistence.

In experiment 1, we showed that the freezing levels between the TRACE and DELAY groups were equivalent during test (Fig. 2*C*, left panel). This behavioral result was inconsistent with previous literature, i.e., generally lower freezing levels following trace conditioning compared with delay conditioning (Raybuck and Lattal, 2014). Different from experiments 2–5, only three CSs were given in this experiment on day 2 because the number of tones presented would initiate different processes. Brief re-exposure of the CS tends to engage memory retrieval followed by reconsolidation, while prolonged re-exposure engages extinction (Merlo et al., 2014; Cahill and Milton, 2019). Thus, the brief re-exposure in experiment 1 ensured that c-Fos expression corresponded to fear retrieval, but not fear extinction (Bouton, 2004). The weaker CS-US association by trace procedure may require more trials to emerge. Indeed, when 10 CSs were presented during the retrieval tests (experiments 3 and 4), DELAY groups showed stronger CS-US associations compared with TRACE groups (significant main effect of group), and there was a faster decline of freezing levels in TRACE groups (significant interactions between group and trials).

During the encoding phase in experiment 1 (Fig. 2*D*, upper panel), the UNPAIRED group showed up-shifts in the c-Fos expressions compared with NoCOND group. Several factors may have contributed to the result. For example, earlier studies have shown that encoding of contextual fear is ReRh dependent (Ramanathan et al., 2018a). Moreover, increased c-Fos expression was observed in the Re when rats were subjected to peripheral noxious stimulation (Bullitt, 1990). However, we did not have the animals that underwent “contextual fear conditioning procedure” (i.e., no tones during conditioning) or “shock-only procedure” (i.e., animals received footshocks in another novel context) in this study. Without these two critical control groups, we cannot rule out the possibility that the increase in c-Fos expression was a consequence of contextual fear encoding or a consequence of footshocks. Nonetheless, such increase in c-Fos expression in the UNPAIRED group did not reach significance in any of the coronal levels we examined (all *p*s > 0.05). This result suggested a pivotal role of the CS-US temporal contingency in recruiting the Re during encoding of fear memory. We also acknowledged the limitation that there were no other control brain regions reported in this study. Thus, we could not conclude whether there was a general up-shift in c-Fos expression at the circuitry level.

Many past studies considered the unpaired procedures as behavioral controls to demonstrate that animals would not acquire CS-US associations under such training procedure (Gormezano et al., 1982; Papini and Bitterman, 1990; Burman et al., 2014). Interestingly, our data showed that the UNPAIRED animals demonstrated divergent high or low freezing levels during tone test (Fig. 2*C*). What CS-US relationship the animals acquired, despite that the CSs did not explicitly predict the occurrence of USs in unpaired procedures, awaits further identification. We suspected that the UNPAIRED animals in our study may have adopted different strategies to cope with an upcoming threat, the US. Some animals may have still acquired the CS-US association despite the long trace intervals, and thus demonstrated high freezing levels during CSs. Since the Re was not required during retrieval after the animals have acquired the trace fear, low c-Fos expression was observed, in line with the DELAY and TRACE groups. On the other hand, some animals may have instead considered the CSs as safety signals, in that with such long trace intervals, the CSs signal the absence of the USs for a certain period of time (Moscovitch and LoLordo, 1968; Kalat and Rozin, 1973). Under such scenario, the animals showed low fear during the CSs at the behavioral level. However, the relatively high c-Fos expression indicated the recruitment of the Re neurons at the neurobiological level. Indeed, earlier studies have shown that theta activity in the HPC differed when the animals were exposed to danger and safety signals (Kasicki et al., 2009) and that the circuit of mPFC and basolateral amygdala was dynamically engaged in fear discrimination between CS− (safety signal) and CS+ (Likhtik et al., 2014). The Re, which has reciprocal projections with the HPC and the mPFC, is therefore likely to participate in the coordination of the neurocircuitry of safety signals. It is worth noticing that the context and the CS always compete for associative strength with the US (Phillips and LeDoux, 1994; Marchand et al., 2014). Phillips and LeDoux suggested that in the procedures with paired CS-US associations, the phasic tone CS is the primary stimulus that associates with the US with contextual cues occurring in the background. On the contrary, when there is no phasic CS that is explicitly paired with a US, i.e., unpaired procedure, the primary associations are between the US and static contextual stimuli, which are therefore in the foreground (Phillips and LeDoux, 1994). Their results, together with Marchand et al. (2014)’s findings, demonstrated that animals in the unpaired group froze less during the CS test and more during context test when compared with paired groups (Phillips and LeDoux, 1994; Marchand et al., 2014). However, in this current study, we did not have an independent context test back to the original conditioning context to examine such scenario.

A recent study suggested that the ReRh is essential for hippocampal-dependent encoding of “precise” contextual memories and that the memory deficit induced by ReRh inactivation could be rescued when the ReRh was also off-line during retrieval test (Ramanathan et al., 2018a). The authors proposed that animals without a functional ReRh could acquire an “elemental” representation of the context and associate it with the USs using a non-hippocampal system. Associations based on only one or few salient features of the behavioral procedure may result in difficulty to discriminate different contexts, and therefore more likely to generalize fear outside the conditioning context. Since the ReRh serves as a critical node between the hippocampal system and the mPFC to support trace fear learning, we examined whether bringing the ReRh off-line during the entire process of trace procedure may also lead to reprised trace fear during retrieval. Indeed, freezing levels of these animals remained high throughout the test session from BL to the last trial (MUS-MUS group; Fig. 6*C*, right panel). The results are likely a combined effect of generalized fear to context and tones without a functional ReRh during the trace procedure. High freezing levels during BL were consistent with earlier studies (Xu and Südhof, 2013; Ramanathan et al., 2018a) that the animals were less capable to differentiate contexts and showed fear generalization to a novel context. Moreover, these animals were also slower in the decline of their freezing levels to the tones compared with controls at the later trials. Different from the V-TRACE group in experiment 2, the SAL-SAL group expressed faster decline in freezing levels. This result could be attributed to different cohorts of animals being used, and to the non-specific effect caused by our manipulation, i.e., two infusions in experiment 5. Additional experiments to dissociate the relative contribution of fear generalization to contexts versus trace fear to tones are needed. Nonetheless, the ReRh serving as a node of the mPFC-HPC circuitry may be critical in both precise encodings of contextual and temporal associations.

In summary, the findings that the ReRh serves as a central character in encoding the trace fear may provide crucial insights into the research regarding fear-related mental illnesses. Trace fear conditioning serves as a suitable model to conceive human emotional learning as human fear learning sometimes involves a trace temporal interval. Moreover, because this procedure recruits higher order nervous system (Bangasser et al., 2006; Gilmartin and Helmstetter, 2010), trace fear conditioning is also used to study cognitive functions. In recent decades, trace conditioning has been adapted to screen for cognitive disruption in mouse disease models for schizophrenia (Brzózka and Rossner, 2013) and Alzheimer’s disease (Kaczorowski et al., 2011). Regarding the multiple roles of the ReRh in fear conditioning, depression, and stress-induced behaviors (Kafetzopoulos et al., 2018), research looking into the ReRh and underlying neurocircuitry of trace fear conditioning will provide a further understanding of fear behavior and mental disorders.

## Synthesis

Reviewing Editor: Macià Buades-Rotger, University of Luebeck Decisions are customarily a result of the Reviewing Editor and the peer reviewers coming together and discussing their recommendations until a consensus is reached. When revisions are invited, a fact-based synthesis statement explaining their decision and outlining what is needed to prepare a revision will be listed below. The following reviewer(s) agreed to reveal their identity: Jean-Christophe Cassel, Mathieu Wolff.

Dear authors,

Thank you for your article on the role of the nucleus reuniens (Re) in the encoding of trace fear memory. The topic is timely as this region is relatively understudied but indeed attracts now wide interest. The design is clean and straight forward and employs both correlative and experimental data. The systematic inactivation approach is a strength: it produces useful results, generally in line with already published evidence. That being said, there are a number of important concerns at the behavioral and analytical levels which undermine some central claims, as detailed below.

MAJOR CONCERNS:

1) Ethical aspects: for perfusions, rats were deeply anesthetized with CO2. This is a short-lived anesthesia which might not be compatible with the delay required to start an intracardiac perfusion. In the rats that were killed, it should be mentioned that rats were dead when perfusion was started. Furthermore, please indicate how many rats have been used in this series of experiments (the total), including the ones with canula misplacements. Also, please indicate exactly how many died and how many were killed. In general, the authors should give much more detail about the procedure (duration of exposure to CO2, delay before starting the preparation of the rats, latency to the start of perfusion...). Has this particular aspect of the methods used in these experiments also been validated by an ethical committee? Again, should the reader understand that the perfusion was started when the rat was dead? If so, please state it clearly. Also, please change the word “sacrificed” to “killed” throughout the manuscript.

2) In the introduction, lines 55-59, the authors claim that the mPFC and the hippocampus are both necessary for trace conditioning. To support this, they cite two articles: Bangasser et al., 2006, in which only the hippocampal function has been altered, as well as Gilmartin and Helmstetter, 2010, who showed that trace conditioning required glutamatergic neurotransmission in the MPFC. The same group however (Gilmartin et al., 2012) showed that muscimol inactivation of the MPFC had no effect on trace conditioning. It is not acceptable to construct an experimental rationale on such a partial consideration of the literature. If data are contradictory regarding the implication of the mPFC in trace conditioning, this does not provide a strong a priori support to the aim of this study. Is the mPFC involved or not? Gilmartin answer ‘yes’ and ‘no’. The authors should acknowledge and discuss the inconsistencies in the literature, and perhaps try to find other references (maybe the human neuroimaging literature helps?) that substantiate the present study.

3) Please clarify the rationale and the associated hypotheses for experiment 5.

4) How did the authors ensure that no surrounding structures were damaged by the inactivation procedure? Figure 1C shows that inactivation was indeed really specific to ventral midline thalamic nuclei. If that image is representative, then it shows that Re inactivation is subtotal (the lateral portions were not affected) and that the rhomboid nuclei was affected to a similar extent. If so, the authors should report the inactivation as “ReRh", as it is almost impossible to target one without hitting the other. Moreover, the authors should clarify that the inactivation was *subtotal* throughout the paper.

5) In lines 65-68 the authors broadly state that Re is “involved” in many functions. The authors should briefly explain what the precise role of Re in these processes is, and if, perhaps, there is a common computation to all of them. Maybe review articles might help? Also, please change the term “spatial working memory” to “spatial navigation", as it is not clear whether general and spatial working memory are separate processes or constructs.

6) The conditioning procedure used was intense: 10 footshocks at 1mA. As a result, very high levels of freezing are obtained throughout all experiments even after only 3 shocks, thus resulting in large overtraining (which the authors do mention briefly in lines 478-479). As a result in all experiments there is virtually no evidence of extinction during the tone test without shocks (except in the final expt for the SAL-SAL group). This impacts the interpretation of the results regarding Re involvement, and deeply affects the whole c-fos study because there are no controls that only received shocks (there is one that only received the CS). It is thus possible that Re recruitment is determined by shock occurence rather than learning per se, and thus its activation reflects nociception or some other process. If we sum up the data, all groups including the unpaired group were found to show higher c-fos activity after conditioning (during which animals experienced shocks) whereas there was pretty much no increase after the test (during which there was no shock). What can the authors say regarding these puzzling results? This should also be discussed at length in the Discussion, as it is a central limitation to interpret the c-Fos results as supporting a role of Re in fear conditioning.

7) The behavioral procedure for the test was inconsistent with other experiments (only 3 CS for this specific experiment, while 10 were presented for inactivation experiments). Can the authors justify this this discrepancy?

8) Did the authors also pay attention to c-Fos expression in other regions such as e.g. the mPFC and hippocampus? Did they look at what had gone on in the rhomboid nucleus, which is connected with the amygdala, or in the amygdala itself? mPFC and hippocampus data should be reported if the authors have it, along with the already reported results. Differences in c-Fos expression between unpaired, delay and trace rats could appear at circuit level, rather than in only the reuniens nucleus. Importantly, there were no control regions. In absence of data from other regions, this major limitation should be discussed.

9) Interactions are not reported for experiment 2 during the critical test. We need to know at least if the critical GROUP*DRUG interaction approached significance because it is the one test to address whether Re specifically supports trace fear conditioning or not. Depending on the results of these interactions, the interpretations may need to be toned down accordingly. It is strange that this is not reported here while it is for other experiments. With such a design we expect to know about all interactions for all experiments. These are the critical data. Please add this much needed information throughout all experiments, in a systematic manner.

10) Also, and importantly, please provide some measure of effect size alongside p-values for all pairwise comparisons, at the very least t-values and degrees of freedom.

11) The authors should report how they statistically analyzed the c-Fos data, as it is only explained how the behavioral data was analyzed.

12) Fisher LSD test, even when corrected, is not very conservative. Why did the authors use this test and not a more conservative one (e.g., Newman-Keuls, Tukey ...)? In most experiments, the group sizes were rather small and thus a conservative correction would be in order, except if there is some reason to do otherwise.

13) The behavioral data are not always consistent. Quite often, the trace condition appeared to yield slightly lover freezing but it is not always the case and experiment 1 is a good example. The authors interpret their results as consistent with the literature when it is the case (experiment 2, e.g. line 304), but then omit to do so where relevant (at least in experiment 1, e.g. line 245). Again, the authors should address the discrepancies in the literature rather than discussing them selectively.

14) Some of the main conclusions of the study are unsubstantiated. For instance, the Discussion starts with “In this study, c-Fos expression first revealed that the Re was generally recruited in the encoding, but not the retrieval phase, of both the delay and trace fear conditioning.” Keeping this statement as it stands would suppose that unpaired rats had lower c-Fos levels when compared to delay and trace rats. This was not the case, perhaps due to the reasons explained in point 6. Thus, the authors must tone down most of the conclusions regarding the involvement of Re in encoding fear conditioning and add the necessary notes of caution. Also, as explained in point 9, claims regarding the necessity of Re for trace fear conditioning should also be toned down depending on the statistical size and significance of the critical interaction. The discussion should thus in general be truer to the data, as eneuro is all about honest reporting.

15) The final experiment apparently aimed to assess whether this effect was “state-dependent” but the results are difficult to interpret because Re inhibition during both encoding and retrieval resulted in high and generalized freezing behavior. The authors should thus tone down the alleged “state-dependent” role of Re and address this in the Discussion.

16) In general, there were few differences between TRACE and DELAY groups. Would the authors not expect a difference between these groups? The authors should address this gap between observed data and theory in the Discussion.

MINOR CONCERNS

17) The authors were likely not aware of this work but there is a new reference which is highly relevant to the present work as it also focuses on RE and fear memory with both c-fos and inactivation approaches: Quet et al., BSF 2020. Most likely the authors would like to consider it for discussion.

18) Consolidation. The authors could distinguish more clearly between fast consolidation (presumably the one they examine) and slow, system-consolidation (over weeks). The Loureiro et el. 2012 paper mentioned by the authors falls into the latter category and it is not adequately discussed (line 473, only remote, not recent spatial memory is affected, so the critical factor is time, not spatial memory per se). Considering the Vetere et al., 2017 paper could be off interest as well (system-level, contextual fear consolidation over weeks).

19) With an unpaired procedure, both the CS and the context compete for associative strength, contextual conditioning thus clearly prevails but conditioning to the cue develops as well, even if this is only at a low extent (e.g. Marchand et al., 2014). Unpaired conditioning should not be considered only a control but rather as a way to assess background versus foreground conditioning (e.g. Philips & Ledoux, 1994). Please comment on this in the Discussion.

20) Line 163: ‘accumulated’ is probably not a correct word. What about ‘accustomed’ or ‘habituated’?

21) Experiment 3: the freezing curves are a bit different from those of experiments 2 and 4. What may it mean? The same remark applies to experiment 5, in which MUS-SAL rats seem to exhibit extinction over time. This might deserve consideration in the Discussion.

22) Lines 311-314: this belongs in the Discussion, not here.

23) Lines 329-336 are confusing. Please rewrite more clearly.

24) Lines 339-343: the control groups were merged despite having different experiences. Why did the authors do so? What is the outcome of the statistical analyses when the groups are not merged?

25) Why were the groups so small? (this is a general remark except for experiment 5, in which groups include 9 or 10 rats). Is it a matter of canula misplacement?

26) Line 484: what is meant by ‘late phase consolidation’? Does it refer to a few hours or a few days, or even more, following task encoding? This concept should be clarified, adding references if needed.

27) Please indicate figure numbers on the figures.
